# Advances of Cobalt Nanomaterials as Anti-Infection Agents, Drug Carriers, and Immunomodulators for Potential Infectious Disease Treatment

**DOI:** 10.3390/pharmaceutics14112351

**Published:** 2022-10-31

**Authors:** Yuhe Ma, Wensen Lin, Yongdui Ruan, Hongmei Lu, Shuhao Fan, Dongsheng Chen, Yuhe Huang, Tangxin Zhang, Jiang Pi, Jun-Fa Xu

**Affiliations:** 1 Guangdong Provincial Key Laboratory of Medical Molecular Diagnostics, The First Dongguan Affiliated Hospital, Guangdong Medical University, Dongguan 523808, China; 2 Institute of Laboratory Medicine, School of Medical Technology, Guangdong Medical University, Dongguan 523808, China; 3 Dongguan Key Laboratory of Environmental Medicine, School of Public Health, Guangdong Medical University, Dongguan 523808, China

**Keywords:** cobalt nanomaterials, anti-infectious agents, drug carriers, immunomodulators, anti-infection therapy

## Abstract

Infectious diseases remain the most serious public health issue, which requires the development of more effective strategies for infectious control. As a kind of ultra-trace element, cobalt is essential to the metabolism of different organisms. In recent decades, nanotechnology has attracted increasing attention worldwide due to its wide application in different areas, including medicine. Based on the important biological roles of cobalt, cobalt nanomaterials have recently been widely developed for their attractive biomedical applications. With advantages such as low costs in preparation, hypotoxicity, photothermal conversion abilities, and high drug loading ability, cobalt nanomaterials have been proven to show promising potential in anticancer and anti-infection treatment. In this review, we summarize the characters of cobalt nanomaterials, followed by the advances in their biological functions and mechanisms. More importantly, we emphatically discuss the potential of cobalt nanomaterials as anti-infectious agents, drug carriers, and immunomodulators for anti-infection treatments, which might be helpful to facilitate progress in future research of anti-infection therapy.

## 1. Introduction

Infectious diseases are the second most important cause of human death worldwide [1]. Main symptoms of infectious diseases include fever, increased pulse, increased breathing, anxiety, and insanity, which may lead to rapid death in some uncontrolled infection conditions. Antibiotics are the cornerstone of therapy for infected critically ill patients, and have saved millions of lives worldwide. However, antibiotics are often not optimally administered due to the compliance of patients, which always results in less favorable patient outcomes and drug resistance [2]. Recent years, drug resistance to some commonly used antibiotics has been widely developed with the abuse of antibiotics, which further increases the emergence of the extremely dangerous multidrug-resistant mutant [3]. Therefore, it is urgent to explore new therapeutic strategies for more effective control of infectious diseases.

Cobalt (Co) and its compounds are widely distributed in nature with numerous anthropogenic activities [4]. Cobalt is considered to be an essential trace element as it is a critical component of vitamin B12 [5]. Cobalt can regulate the metabolism of fatty acids and affect the synthesis of amino acids and proteins in nerve cells. Moreover, cobalt compounds also have promising medical applications. For example, cobalt-based alloys can be used to make frictional and supporting parts of artificial joints, such as femoral heads, acetabular liners, femoral components, and stems due to their excellent corrosion and wear resistance, strength, and machinability [6].

Nanoparticles (NPs), typically defined as ultra-small particles ranging from 1 to 1000 nm in diameter, can be comprised of different materials such as lipids, polymers, or metals, all of which offer unique delivery advantages [7]. During recent decades, metal nanoparticles (MNPs), one of the most important catalysts for biomedical application, have gained increasing attentions [8]. Among these MNPs, cobalt nanoparticles (Co NPs) are not only used in engineering fields, but also in the medical area according to their advancing properties [9]. For example, one study showed that Co NPs have the ability to induce cell apoptosis [10], which indicates the feasibility and relevance of Co NPs as novel nanomedicines for potential chemotherapy, phototherapy, and thermotherapy.

Cobalt can strengthen protein metabolism and promote the synthesis of some enzymes, thus can enhance the body immunity of human. Based on the promising properties of cobalt, Co NPs are also used as potential therapeutic agents for the treatment of various infectious diseases [11,12]. Co NPs are known to induce the production of reactive oxygen species (ROS), which are responsible for the inhibition effects of Co NPs against different kinds of bacteria, fungi, and viruses [13,14]. Here, we summarized the biological activity and biomedical uses of Co NPs, especially their potential for anti-infectious treatment, which may contribute to the future development of anti-infection strategies.

## 2. The Biological Activity of the Cobalt Element

### 2.1. Cobalt Is the Core Element of VB12

**Figure 1:**
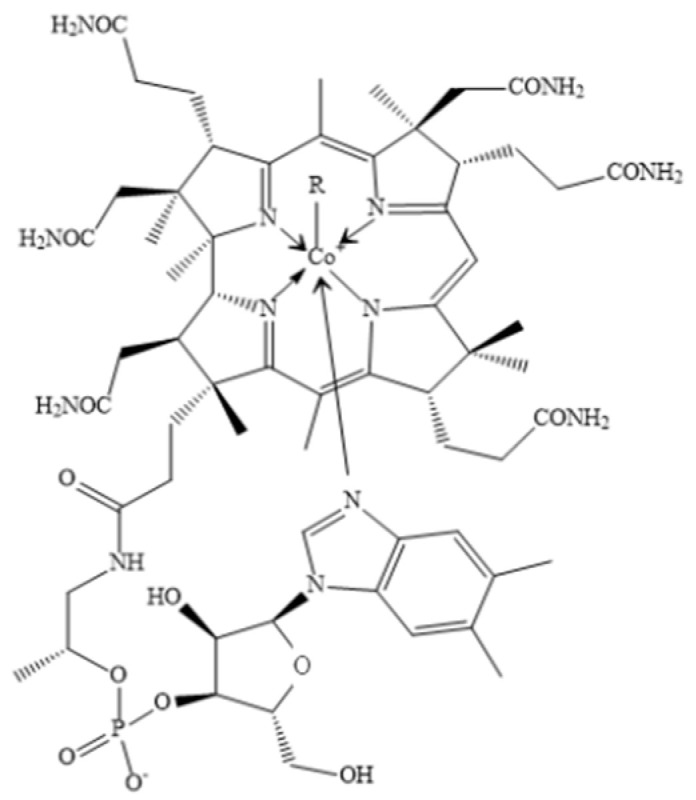
The chemical formula of VB12. R = 5′-deoxyadenosyl, CH3, OH, CN.

Cobalt, a silvery-gray, lustrous, brittle but hard metal, is distributed widely in nature, including rocks, soil, plants, and animals. As a transition metal located in the fourth row of the periodic table, cobalt is a neighbor of iron and nickel with an atomic weight of 58.9 [15]. Cobalt is an essential trace element for human health and can occur in organic and inorganic forms. The most widely known organic forms are the core element of cobalamin (vitamin B12 and its derivatives), which serve as cofactors of a wide range of enzymes and components of proteins [16]. As a water-soluble vitamin, vitamin B12 contains mineral cobalt, which is positioned centrally and coordinated with upper and lower ligands as a corrin ring [17,18] (Figure 1). For this reason, the compounds with vitamin B12 activity are always called as “cobalamins”.

### 2.2. Physiological Function of Cobalt Based on Their Critical Roles in VB12

As an essential vitamin, vitamin B12 is obtained entirely from the diet. It is naturally found in animal food products, including meat, poultry, shell, fish, eggs, milk, and other dairy products [19]. It is also synthesized naturally by some large intestine-resident bacteria in humans in the rumen from cobalt and has a major role in metabolism, especially in the peri-parturient period [20,21]. Vitamin B12 is always bound with proteins in food, which must be released by gastric acid breakdown in the stomach, where the salivary R-binder can bind with VB12 to prevent VB12 destruction [22]. The absorption of vitamin B12 into the blood stream is dependent on their binding with the protein intrinsic factor (IF) to form the VB12-IF complex in the small intestine [23].

Vitamin B12 is stored primarily in the liver and always acts as a cofactor for methionine synthesis from homocysteine and succinyl-CoA synthesis from methylmalonyl-CoA in mammalian systems [24]. As an essential nutrient for folate metabolism and DNA synthesis, VB12 is critical for normal fetal and childhood growth and development [25]. Maternal VB12 deficiency during pregnancy may increase the risk of neural tube defects and brain development retardation, as well as preterm birth and low birth weight [26]. VB12 is also necessary for basic body functions, such as the nervous system, cardiovascular system, and immune system [27], as well as the maintenance of skeletal muscle and neurobehavioral parameters, and modulation of gut microbiota [28]. VB12 deficiency has also been associated with several metabolic disorders such as macrocytic anemia, cardiovascular, cerebrovascular, and neurological disorders [29]. Clinical disease caused by VB12 deficiency usually results from the failure of the gastric or ileal phase of physiological B12 absorption, best exemplified by the autoimmune disease, pernicious anemia [30].

### 2.3. The Physiological Function of the Cobalt in Hematopoiesis

As the central cofactor of vitamin B12 and the critical roles for proper nucleotide synthesis, cobalt can also stimulate the hematopoietic system of human bone marrow, which promotes the synthesis of hemoglobin and increase the number of red blood cells [31]. The detailed mechanisms about cobalt stimulated hematopoiesis are summarized as following:

Firstly, as mentioned above, as the active center of the VB12, cobalt participates in the metabolism of ribonucleic acid and hematopoietic substances through VB12, which act on the hematopoietic process [32]. Deficiency of cobalamin (vitamin B12) can result in megaloblastic anemia due to the inhibition of DNA synthesis caused by decreased availability of purines and pyrimidines (Thymidine monophosphate), which results in enlarged red blood cells and accumulation of significant, immature precursors (megaloblasts) of RBCs in the blood and bone marrow [33]. 

Secondly, cobalt is involved in metabolism modulating transcriptional activator hypoxia-inducible factor-1 (HIF-1), which stimulates erythropoietin (EPO) production [32]. HIF-1 is a transcription factor that controls hypoxia-induced autophagy by upregulating the expression of its downstream proteins [34]. Cobalt can activate HIF-1 at normal oxygen levels, which is stabilized, translocated to the nucleus and then dimerized with the constitutively expressed HIF-1 to elicit the transcription of target genes necessary for increased oxygen demand [10]. Study has also demonstrated that cobalt treatment may increase hypoxic tolerance of different tissues, improve muscle metabolism and exercise performance [35]. 

Third, cobalt can promote the absorption of iron. Fe is an essential element important in a wide variety of metabolic processes, including oxygen transport, DNA synthesis, and electron transport. Fe is required for the production of red blood cells and forms part of hemoglobin, helping in the binding and transportation of oxygen in the body [36]. Cobalt can promote the absorption of iron in the intestinal mucosa and accelerate the storage of iron into the bone marrow, which therefore very important for hematopoiesis.

### 2.4. The Anti-Infective Activity of Cobalt

In recent decades, an increasing number of studies have focused on investigating the structure and chemical behavior of some metal compounds to discover new drugs with antibacterial capabilities. Among them, cobalt has proven the ability to act as a potential candidate for antibiotic [37]. Cobalt alloys have high corrosion resistance with a balance among biocompatibility and mechanical strength [38], which makes it suitable for the artificial joint materials manufacturing. It was also recently reported that in addition to inducing a hypoxic response, Co(2+) incorporation could also improve the antibacterial ability of titanium-based bone implants, which suggested that Co(2+) had an additional effect as an antimicrobial agent.

Co^2+^ can directly bind to the DNA of bacteria to induce bacterial cell death by different pathways [39], for example, by inducing reactive oxygen species (ROS) production. ROS is the reduction products of oxygen, such as peroxides, which can destroy the cell membrane of bacteria and play a significant role in DNA and other cellular damage [40]. Apart from that, cobalt can inhibit the function of RecBCD, which is crucial for initiating the SOS repair. The SOS response can promote the integrity of DNA, it also includes error-prone factors that allow for improved survival and continuous replication in the presence of extensive DNA damage [41]. Moreover, cobalt can also be helpful to cure infectious diseases [42], which is also partially associated with the immunological regulation effects. Cobalt can help in the creation and repair of the myelin sheath, which encircles the nerve cells and further protects them from external damage. A study has shown that cobalt is able to induce new blood vessel formation, as well as to improve wound closure and avoid bacterial infection [43].

### 2.5. The Immunoregulatory Role of Cobalt

The immune system, which is integrated into all physiological systems and critical for human health, protects the host against infections [44] and provides constant surveillance of native cells that may be harmful, such as cancerous cells [45]. It is reported that metals are critically implicated in regulating both the innate immune sensing of and the host defense against invading pathogens [46], which suggests that metals play an important role in regulating the immune system against infection. A recent study has shown that transition metal-based compounds could modulate autophagy, one of the most important host immunological responses, which therefore provides a new therapeutic strategy based on transition metal-based compounds for disease treatment [47]. For example, CoCl_2_ treatment activates autophagy through the target genes induced by HIF, and correlates with the expression of certain pro-apoptotic factors [48]. Additionally, Co(2+) can induce an HIF-1α-dependent metabolic shift from oxphos towards glycolysis in macrophages, which plays an early and pivotal role in the inflammatory responses [49]. Thus, the growing interests of researchers in transition metal-based compounds is not only due to their potent antibacterial, antifungal, antiviral, antitumor, and anti-inflammatory properties [50], but also due to the promising immunological regulation effects.

**Figure 2:**
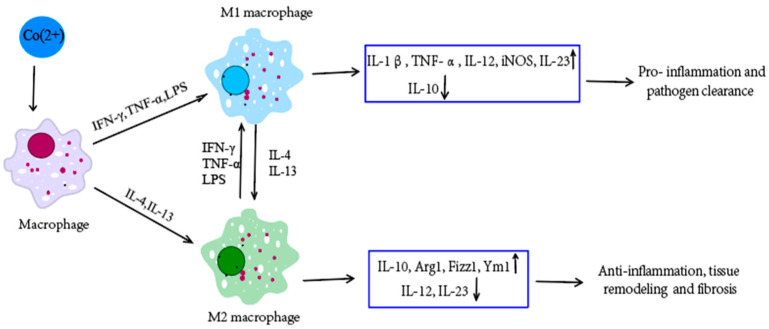
Co(2+) can induce inflammatory responses in macrophages [53]. Different environmental factors can induce the differentiation of macrophages into different subsets: M1 and M2 macrophages, which have different phenotypes, secrete different cytokines, and have different biological activities. M1 macrophages cells are activated by the classical way (activators include IFN-γ, TNF-α and LPS, etc.), which mainly play a role in killing microorganisms and promoting inflammation. M2 macrophage cells are activated by alternative ways (activators include IL-4 and IL-13), which are mainly involved in immune regulation, inhibition of inflammation, and tissue repair, and are related to the chronic progression of infectious diseases. M1 and M2 macrophages can transform into each other in different pathological processes and microenvironments. ↑ means upregulate and ↓ means downregulate.

One study shows that Co(2+) has a significant influence on osteoblastic activity, differentiation, and inflammatory processes [51]. The formation of Co NPs in the wear process of MoM hip implants may lead to inflammatory fluid collections or osteolysis [4]. In vitro studies have shown that Co(2+) can activate the production of bone-resorbing cytokines through the activation of redox-dependent mechanisms and activate the biosynthesis of inducible NO synthase and pro-inflammatory interleukins in macrophages [52], thus inducing inflammatory responses in macrophages [53] (Figure 2). Macrophages are the predominant immune cells in periprosthetic tissues, which induce a type IV hypersensitivity reaction. Both T-lymphocytes and B-cells (to a lesser extent) are involved in the production of inflammatory mediators. Pro- and anti-inflammatory cytokines, the interleukins IL-1, IL-6, IL-4, and tumor necrosis factor-α (TNF-α), play a central role in the inflammatory reaction [54]. A study identified a new downstream effect of cobalt-induced ROS production, which reduced RhoA expression in modulating macrophage migration and cytoskeleton organization, leading to an enhancement in macrophage spreading, adhesion, and inhibition of migration. These effects could induce a prolonged immune cell retention, which thereby propagates the chronic inflammation [55]. Co(2+) can also promote pro-inflammatory gene expression by binding to the human TLR4 receptor [56], which could activate dendritic cells migration to draining lymph nodes and present allergen-induced epitopes to trigger antigen-specific T cell proliferation, differentiation, and memory formation [57]. Co(2+) has also been shown to increase the binding of T lymphocytes to endothelial cells and the transendothelial migration of these lymphocytes [58], which independently induce circulating cytokine or chemokine production to promote the accumulation of T lymphocytes [55]. Moreover, the fibrotic response could be further enhanced by the presence of macrophages. In the light of these results, Co(2+) and macrophages act synergistically to influence the functional properties of fibroblasts and extracellular matrix (ECM) homeostasis [59]. Cobalt can also modulate immune cell functions in the lung and induce airway hyperreactivity with a mixed neutrophilic and eosinophilic inflammatory responses, which are accompanied by dendritic cells and innate lymphoid cells [60]. However, epidemiological studies have demonstrated a higher risk of asthma in workers exposed to cobalt, and several case series of cobalt-induced asthma have also been reported [60], which also highlight the toxicity issue of cobalt to human health.

In summary, as an essential trace element of the body, cobalt has an important physiological role. It is a component of vitamin B12 and some other enzymes, participates in the metabolism of the human body, and has the effect of stimulating hematopoiesis in various ways. The release of cobalt into the human body can also trigger the body’s immune system, which provide new therapeutic strategies for infectious diseases.

## 3. The Synthesis of Cobalt Nanomaterials

Along with the time, various chemical and physical synthesis methods of cobalt nanomaterials have emerged [61], such as thermal decomposition, hydrothermal synthesis, chemical wet processing, thermal reduction, micro-emulsion, precipitation, sol–gel, microwave-assisted, reverse micelles, evaporation–condensation, and laser ablation [62]. Cobalt nanomaterials are always synthesized by top-down or bottom-up methods [63]. In the top-down method, cobalt compounds based on bulk materials are transformed to NPs through sputtering techniques, grinding, and milling. While in bottom-up methods, the self-assembly of miniature compounds into NPs is performed [64]. A typical feature of physical methods for cobalt nanomaterial preparation is the production of particles by the so-called “top-down” approach, such as laser ablation [65], which is opposite to the chemical methods characterized by a “bottom-up” approach. These physical and chemical methods used for cobalt nanomaterial preparation showed a narrow range of size and controlled morphology, which are very suitable for the industry production of cobalt nanomaterials [66]. However, the use of physical methods always requires high temperature or pressure, and most chemical methods need some chemicals that are toxic and hazardous to the environment and biological systems [67]. Therefore, there is still a demand for the development of safer, greener, eco-friendly, and cost-effective synthetic methods that can eliminate the arduousness and complications of current physicochemical methods for cobalt nanomaterials [9].

In the current scenario, green chemistry is known as an intellectual approach for nanomaterial preparation. The surging popularity of green methods has triggered the synthesis of Co NPs using different sources, such as bacteria, fungi, algae, and plants, resulting in large-scale production with less contamination [68]. The bacterial synthesis of nanoparticles has been adopted due to the relative ease of manipulating the bacteria [69]. The fungi-mediated approach exhibits unique advantages, as the growth process of fungi is easily handled and isolated, with the large amount of biomass and high yield of proteins [64]. Compared with bacteria and fungi-based cobalt nanomaterial preparation methods, plant extracts have been extensively used to synthesize Co NPs as it is an inexpensive, biocompatible, and easy scale-up method that can fully avoid the requirement of additional stabilizing agents during the nanoparticle synthesis [70]. For example, IsmatBibi et al. fabricated cobalt–oxide nanoparticles using Punica granatum peel extract from cobalt nitrate hexahydrate at low temperature [71]. Furthermore, the nanoparticles obtained from plant extracts exhibit greater reduction and stabilization effects, which therefore allows the cobalt nanoparticles to show multiple properties, including catalyst/photocatalyst, magnetic, antibacterial, anticancer, and gas sensing [72].

## 4. The Characteristics of Cobalt Nanomaterials

Nanomaterials, due to their excellent physical and chemical characteristics, have become one of the most rapidly growing research areas in the biomedical field in recent years [73]. In broad terms, nanomaterials are inorganic, organic, or polymeric materials that possess physicochemical features with a size range of 1–1000 nm [74]. The small size of nanomaterials allows them to easily distribute throughout the body, traverse biological barriers, and enter the systemic circulation [75]. Nanoparticles always show large surface areas, which therefore can help them interact with biological systems more precisely [76]. In addition, nanomaterials can be engineered to show different properties such as size, shape, charge, and surface chemistry [77], which could lead to different applications in the biomedical fields [78]. For example, nanomaterials allow molecular scale detection for the diagnostic application of pathogenic microbes [79]. Additionally, small-sized nanomaterials always exhibit enhanced permeability and retention (EPR) effects in tumors, with relative increases in local tumor concentrations of contrasting agents [80]. Such promising properties therefore make nanomaterials potential candidates for novel diagnostic and therapeutic method development.

There are various kinds of nanomaterials, including metal nanomaterials, ceramic nanomaterials, carbon-based nanomaterials, polymeric nanomaterials, and lipid-based nanomaterials [75]. Among them, metal nanomaterials are widely used due to their low toxicity, biocompatibility, and excellent conductivity [81]. For example, MNPs have been largely implemented to facilitate the conjugation with biomolecules for the improvement of biosensors [82]. The NiCo@f-MWCNT nanocomposite has been proved to be a kind of novel sensor with high stability and excellent electrochemical properties [83]. Recently, cobalt nanomaterials (Co NMs) have attracted considerable attention due to their low costs in preparation [84], great electrical property, magnetic property, and catalytic property [85]. For example, Mn_0.75_Co_0.25_Fe_2_O_4_ NPs can be utilized in industrial and environmental objects such as water treatment from the pollutant dyes due to their effective photocatalytic performance to protect the environment from toxic pigments [86].

Co NMs are renowned catalysts, particularly in Fischer–Tropsch reactions [87], with high Curie temperature, which makes it applicable over a wide temperature range [88]. The high saturation magnetization and large anisotropy field of pure cobalt nanoparticles give them an intrinsic advantage within the strict requirements of hyperthermia [89]. Co NMs have a variety of size-dependent structural, electrical, magnetic, and catalytic capabilities [90]. Therefore, efforts have been conducted to attain various structures and sizes such as spheres, sheets, snowflakes or cauliflower like particles, and flakes [91]. The instability of Co NMs is their main drawback, which can be diminished by using different stabilizers such as surfactants and dendrimers [92]. Based on these features, Co NMs have demonstrated various biological and medical applications, such as antimicrobial, anticancer, antioxidant, anti-fungal, and enzyme inhibition properties [9].

## 5. Anticancer Activity of Cobalt Nanomaterials and Cobalt-Hybrid Nanomaterials

In recent years, cobalt nanoparticles (Co NPs) have been proven to show promising anticancer activities [84]. Cobalt is a non-accumulative element for the human body [87], indicating that it would not induce metal poisoning caused by cobalt accumulation. Cytotoxicity studies demonstrate that Co NPs exhibit mild anti-proliferative character against the cancer cells and safe nature towards the normal cells [93]. Our recent work also indicated the cancer cell inhibition effects of cobalt oxide nanoparticles (Co_3_O_4_ NPs) with few proliferation inhibition effects on normal cells [94]. The compatibility of Co NPs with human RBC has also been proven to have no harmful effects in the human blood stream [93], which also indicates Co NPs an attractive candidate for cancer treatment.

The anticancer potential of these nanomaterials is attributed to their ability to induce ROS production in cellular compartments, which could eventually lead to the activation of autophagic, apoptotic, and necrotic death pathways [95]. Co NPs can be internalized by cancer cells through endocytosis [87]. After penetrating into the membrane of cancer cells, Co NPs can lead to cancer cell apoptosis [87]. Our recent work indicated that Co NPs inhibited U-87 MG cancer cell proliferation was not cobalt–ion- or apoptosis-dependent, which was due to the ability of Co_3_O_4_ NPs to induce the aggregation of autophagosomes, break the intracellular homeostasis, and block the flux of autophagy [94]. Additionally, Co NPs can also significantly induce reactive oxygen species (ROS) generation, lipid peroxidation (LPO), mitochondrial outer membrane potential loss (MOMP), and caspase-3 enzymes activation in cancer cells [96]. In animal models, Co NPs can accumulate preferentially in cancer sites due to an enhanced permeability and retention (EPR) effect [97], which therefore can passively deliver drugs to tumor tissue [98]. The highly efficacious nanocarriers can ferry cargo such as imaging and therapeutic agents, which make them very suitable for drug delivery as well as diagnosis, facilitating the advent of personalized medicine [99]. By acting as drug carrier, researchers have proved that Co NPs can effectively reverse the side effects of cisplatin [98]. Moreover, drug loading/release characterization reveals that the cobalt nanowires can interact with doxorubicin (DOX) by electrostatic interaction, and accordingly form a composite which can release DOX with a temperature increase under near-infrared light (NIR) treatment [100], which indicates the potential of Co MPs to act as chemosensitizer and protective agents for anticancer treatment. Furthermore, angiogenesis assessment reveals that the released cobalt ion from the nanowires can significantly enhance the angiogenesis efficacy for cancer treatment [100], which provides a promising multifunctional platform for cancer treatment and postoperative recovery. Moreover, we have previously demonstrated that Co_3_O_4_ NPs have shown a very high photothermal conversion rate, which allows the application of Co_3_O_4_ NPs for the photothermal elimination of tumors [94], which allow them to manipulate protein degradation pathways (ALP and UPS) and photothermal therapy for enhanced anticancer treatments both in vitro and in vivo [94]. Additionally, CoNWs-GO-PEG-DOX nanosystems show the satisfactory effect to eliminate cancer cells with synergistic chemo-photothermal therapy in vitro and have the potential to serve as a targeted antitumor agent in synergistic chemo-photothermal therapy [101]. In addition, DAPI stained nuclear DNA staining analysis has demonstrated that Co_0.5_Ni_0.5_Nb_x_Fe_2−x_O_4_ nanosystem can cause nuclear DNA disintegration and programmed cancer cell death [102]. The Co_3_O_4_@Glu/TSC nanoparticles’ complex can also inhibit the growth of cancer cells by inducing apoptosis in them with their anticancer activity [103], while the Co(OH)_2_@Glu-TSC nanosystem can also be considered as a new treatment for breast cancer by inducing cancer cell apoptosis [104].

These results collectively suggest the anticancer application of Co NMs based on their promising biological activities, which also indicates their potential for the treatment of other diseases, such as infectious diseases.

## 6. Anti-Bacterial Activity of Cobalt Nanomaterials and Cobalt-Hybrid Nanomaterials

Bacteria, especially antibiotic-resistant bacteria, are currently one of the most serious issues worldwide, which are responsible for numerous life-threatening diseases [105]. Every year, bacterial infection leads to substantial morbidity and mortality worldwide [106], which requires more effective control of bacterial infection. Cobalt-based nanoparticles (CBNPs) have displayed dose-dependent cytotoxicity and antimicrobial activities against microbial species [107].

Cobalt nanomaterials have shown promise in the antimicrobial applications against a vast diversity of bacteria [37]. Singh has introduced a plant extract-based route for the synthesis of cobalt nanoparticles and their potential anti-bacterial uses [70]. For example, Suvarta D et al. introduced the biogenic synthesis of Cobalt nanoparticles using Hibiscus cannabinus leaf extract, and demonstrated their antimicrobial activity against *Bacillus substilis* and *Escherichia coli* [108]. And Co_3_O_4_ NPs were evaluated against Gram negative and Gram positive bacteria to show active inhibition against *Klebseilla pneumonia* and *Bacillus lichenifermia* [109]. There is also a study showing that Co NPs have even stronger antibacterial activities compared to the standard antibiotic drug ciprofloxacin [110], and the low-concentrated Co NPs are non-toxic in vivo which make thempotential substitutes as novel antibiotics [111]. Moreover, Co NPs have shown amazing antibacterial activity against multidrug-resistant pathogens, including *Staphylococcus aureus*, *Proteus* spp., *Bacillus substilis*, and *Escherichia coli* [37]. Li et al. found that CoFe_2_O_4_ nanoparticles can act on Gram-negative bacteria at lower concentrations [112], and show the ability to attach to negatively charged bacterial cells [113]. Zn_0.75_Co_0.25_Fe_2_O_4_ NPs are reported to replace some disinfectant solutions used for surface washing in hospitals and for inclusion in some paints used in the medical operating rooms to defend the pathogenic microbes [86].

However, the bacterial detection ability of NPs varied depending on the different factors [114], such as bacterial strain, concentration, and particle size. For example, small Co NPs showed statistically higher toxicity compared to large Co NPs under experimental conditions for the bacterial systems [90].

**Figure 3:**
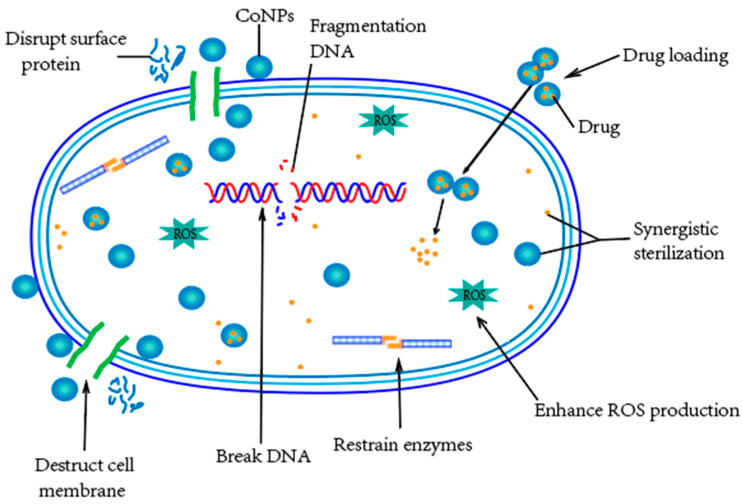
The distinct mechanisms against bacterial infections of cobalt nanomaterials: the surface protein and cell membrane disruption, production of ROS, destruction of DNA, inhibition of enzyme synthesis, delivery of therapeutic agents, and synergistic sterilization.

Additionally, cobalt nanomaterials leverage distinct mechanisms against bacterial infections and the mechanisms are summarized in Figure 3. Cobalt-based nanomaterials can not only act as direct bacterial inhibition agents, but can also act as drug carriers for antibiotics and natural antimicrobial compounds for more effective anti-bacterial treatments [115]. Firstly, nanoparticles (NPs) can serve to reduce the toxicity, enhance the bioactivity, and improve the targeting effects of drugs, which could result in increased drug bioavailability and efficacy with reduced side effects [116,117]. Based on the advantages of nanomaterials, Co NPs allow drugs to reach the infected areas of the body while keeping healthy tissue uninfected. In addition, the magnetic properties of Co NPs can positively affect the targeting effects of drug delivery, which indicates that we can control the targeting effects of Co NPs using an extra magnetic fields [93].

Secondly, the surfaces of the Co NPs can interact directly with the bacterial outer membrane, causing the membrane damage to destroy the bacteria functions and growth [108]. Thirdly, the small Co NPs with a high surface-to-volume ratio interact with the bacteria’s outer membrane and cause a change in its permeability. This higher permeability allows the NPs and the encapsulated drugs to enter the bacteria, which can thereby kill the bacteria more effectively [37]. 

Moreover, the positively charged metal ions from Co NPs, such as Co(2+), can directly interact with the cell surface of Gram-negative bacteria, which is negatively charged at near-neutral pH due to the presence of lipopolysaccharides, potentially leading to inhibition of enzyme biosynthesis [118]. Moreover, due to the optimal and potent antibacterial activity and proper stability of Co NPs [119], it is difficult for microbial pathogens to develop resistance towards them [120]. This property therefore provides new possibilities to develop more effective methods for bacteria killings with the reduced emergence of drug-resistant mutants.

Interestingly, Co NPs can also be used for bacterial detection based on their promising physical, chemical, and biological properties. For example, researchers incorporated a bovine serum albumin-templated Co_3_O_4_ magnetic nanoenzyme with a novel specific fusion phage protein, which could be combined with magnetophoretic chromatography to detect *Staphylococcus aureus* [121]. These results demonstrate the potential of Co NPs to construct novel nanobiosensors for bacterial detection.

## 7. Anti-Virus Activity of Cobalt Nanomaterials and Cobalt-Hybrid Nanomaterials

Viruses are mainly formed by nucleic acids (DNA or RNA) and which can infect their host cells, use parts of the cellular machinery to reproduce, and release the replicated virus to infect more cells [122]. It is widely accepted that viruses are currently the most threatening pathogens to human lives due to the epidemic of COVID-19, which has caused millions of deaths worldwide with more than 5 hundred million infected cases.

Numerous nanomaterials have shown their potential for the control of virus infection [123,124], and among them, metallic nanomaterials are also considered to have a wide variety of activities against viruses [125]. Metal nanoparticles by virtue of their unique shape, size, structure, and local-field enhancement action can interact with viral surface proteins through Kazimir interaction and van der Waals forces causing its inactivation [126], which provides new potential antivirus methods. Interestingly, Co NPs have also been reported to show attractive antiviral properties [127]. Delong et al. indicated the potential anti-virus effects of cobalt-doped ZnO nanoparticles [128], which demonstrated the potential use of cobalt to enhance the anti-virus effects combining with other metal nanoparticles. Kevadiya et al. synthesized a kind of Europium (Eu3+)-doped cobalt ferrite (CF) dolutegravir (DTG)-loaded nanoparticles, and further investigated their use as platforms for nanoformulated drug biodistribution, which might benefit the long-acting, slow, and effective release of antiretroviral therapy by drug delivery to human immunodeficiency virus cell and tissue reservoirs [129]. These results suggest the potential of cobalt to construct anti-viral nanoparticles, although further investigation on the anti-virus activity of pure Co NPs is needed.

**Figure 4:**
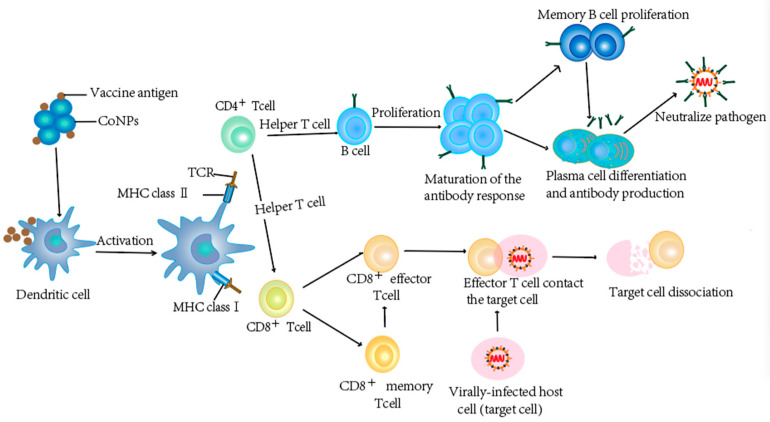
Cobalt nanomaterials can be used for vaccine delivery. Vaccines contain weakened or inactivated parts of specific organisms (antigen) that can trigger an immune response in the body, producing a small amount of antibodies and memory T cells and memory B cells. When the body is exposed to the same antigen again, these two cells quickly proliferate and differentiate to produce effector T cells and effector B cells (plasma cells) to quickly remove the antigen and then: plasma cells produce antibodies and neutralize antigen binding. Effector T cells and the target cells are in close contact, making the target cells lysis and death.

**Figure 5:**
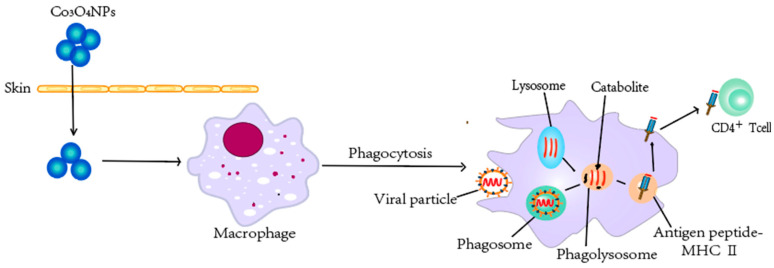
Cobalt nanomaterials help to enhance immunization efficacy. Co3O4NPs can penetrate human skin and introduce considerable immunostimulatory when pulsed with macrophages. Macrophages have powerful phagocytosis, digestion, and killing functions, can intake pathogens such as virtual particles, form phagosomes, and fuse with lysosomes forming phagolysosomes, through oxygen-dependent and oxygen-independent systems, under the participation of a variety of enzymes, and kill and digest pathogens. Additionally, macrophages are an important class of dedicated antigen-presenting cells that can uptake, process antigens, and present antigen peptide-MHC-II molecular complexes to CD4+ T cells.

One of the most effective means to combat virus infections are vaccinations [130], which can be achieved by the development of novel vaccines [131]. Due to the remarkable physical/chemical properties, high surface area to volume ratio, and high drug-loading capacity, nanomaterials can be used for both drug and vaccine delivery [132,133]. The roles of Co NPs are exhibited in Figure 4. Encapsulation or conjugation of antigens within nanomaterials can greatly increase the persistence of antigens at the injection site, in the circulation, lymphoid tissues, or even within antigen (Ag)-presenting cells (APCs) [134]. The promising ability of NPs to act as a cargo of immunogens for modulating immune responses [135], including cell recruitment, activation of APCs, and induction of cytokine and chemokine [136], is very attractive for the development of novel investigations on in vitro immunogenicity of Co_3_O_4_ NPs and their effects on cancer-associated or tolerogenic cytokines [137]. Co_3_O_4_ NPs have been shown to penetrate human skin and introduced considerable immunostimulatory when pulsed with macrophages [137] (Figure 5), which have shown the potential of Co_3_O_4_NP to enhance immunization efficacy. These results collectively suggest that Co NPs can serve as potential antigen carriers for the development of vaccines against viruses, which remains to be further explored.

Finally, cobalt nanomaterial can also be applied to construct nanobiosensors for the detection of viruses. For example, by immobilizing HBV probe DNA (ssDNA) onto Co_3_O_4_ nanostructures through coordinate bond formation between nucleic acid of ssDNA and Cobalt metal, the obtained ssDNA/Co_3_O_4_PNCs/GCE system can act as potential electrode to test HBV DNA in blood serum and urine samples [138]. Azab et al. also introduced a method using Co NPs constructed nanobiosensors for the determination of daclatasvir: a hepatitis C antiviral drug [139]. Co-metal functionalized TiO_2_ nanotube was developed as a sensing material for the electrochemical detection of SARS-CoV-2 infection through the detection of the receptor binding domain (RBD) of spike glycoprotein [140].

These results indicate that Co NPs can be engineered into novel nanobiosensors for the detection of virus or anti-viral drugs, indicating the promising application of Co NPs besides the anti-viral vaccination and treatment.

## 8. Anti-Fungal Activity of Cobalt Nanomaterials and Cobalt-Hybrid Nanomaterials

Fungi are a kingdom of multicellular eukaryotic organisms that are heterotrophs. As an important part of the microbiota in healthy barrier tissues [141], the dysbiosis of fungi would lead to different diseases, which make fungal infections an increasing threat to global public health [142].

Cobalt nanomaterials have also showed various biomedical applications, including anti-fungal uses [84]. Al-Fakeh MS et al. introduced that the cobalt oxide nanoparticles made by calcination method showed stronger anti-fungal activity than the cobalt oxide nanoparticles obtained by other methods due to their small particle size and large surface area to induce the production of ROS [143]. Another study showed the antimycotic efficacy of CoFe_2_O_4_ nanoparticles against *Fusarium oxysporum*, *Colletotrichum gloeosporioides*, and *Dematophora necatrix* [144]. By anti-fungal investigations through colony forming unit (CFU) technique and SEM, Co_0.5_Ni_0.5_Ga_x_Fe_2−x_O_4_ (0.0 ≤ x ≤ 1.0) nanosystem was found to inhibit the growth of *Candida albicans* [145]. The synthesized cobalt ferrite nanoparticles were found to be potent antifungal activities against *Aspergillus niger*, *Alternaria solani*, *Fusarium oxysporum*, and *Candida albicans* [146]. CoO NPs could also be obtained by using natural extracts for phytosynthesis followed by a calcination step (500 °C) to obtain crystalline NPs, which showed antimicrobial potential towards fungi [147]. Additionally, Hasan M et al. also found that Co_3_O_4_ NPs synthesized by *Withania coagulans* using different solvent combination ratios showed different anti-fungal activities. A 90% fraction of hexane/H_2_O showed excellent anti-fungal activity against *P. niger* and *C. albicans*, while 70% methanol/hexane showed strong anti-fungal activity for *C. albicans*, which indicated the potential of Co_3_O_4_ NPs for the treatment of various fungal infections [148]. These results strongly suggest the inherent inhibition effects of cobalt nanomaterials against fungi.

Nanomaterials not only exhibit improved inhibitory activity against fungal pathogens at low concentrations, but can also act as nanocarriers to assist the targeted delivery of anti-fungal drugs [149]. Nanoparticles have the potential to carry, stabilize, and protect therapeutic payloads, which can penetrate extracellular polymeric substances (EPS) for targeted fungal cell killings [150]. Various commercially available anti-fungal drugs can be loaded into nanostructures, which significantly enhance their anti-fungal activities [151]. As a kind of functional nanomaterial with drug loading and delivery abilities, cobalt nanomaterials are expected to further assist anti-fungal treatments serving as drug carrier, which needs to be further investigated.

Most of the pathogenic fungi are opportunistic in causing disease under immunocompromised conditions [152]. Hence, there are significant interests in stimulating the immune system to obtain more effective anti-fungal immunological responses against pathogenic fungi. Co(2+) can induce ROS production and reduce RhoA expression, which could further modulate macrophage migration and cytoskeleton organization. Moreover, these effects on ROS and RhoA cascades could also lead to an enhancement in macrophage spreading and adhesion, and also regulate the inflammatory responses [55]. These effects strongly suggest the potential of cobalt nanomaterials to regulate host immunity for anti-fungal treatments, as the cobalt ions are abundantly involved as byproducts of cobalt nanomaterials.

Currently, although there are limited reports for the use of cobalt nanomaterials, the potential abilities of cobalt nanomaterials to directly inhibit fungal growth, to act as drug carrier and delivery system, and to stimulate anti-fungal host immunity make them potential candidates for more effective anti-fungal treatments.

## 9. Other Biological Applications of Cobalt Nanomaterials and Cobalt-Hybrid Nanomaterials

Apart from the strong anticancer and anti-infection activities, cobalt nanomaterials also have various other biological and medical applications. An increasing number of studies have shown that cobalt nanomaterials have the potential to fight parasitic infections. Khalil et al. proved that Co NPs displayed antileishmanial activity against both the axenic promastigote and amastigote cultures [153], which might be one of the possible options in nanomedicine to treat leishmania at any stage of the life cycle. There are also findings showing the potential of Co NPs against *A. castellanii* due to their significant amoebicidal effects and inhibition of encystation [11]. Cubic CoO NPs with an average size of 20.03 nm diameter have been prepared using the leaf extract of *S. thea* to show strong antioxidant capacity [153]. In addition, green-synthesized CoO NPs [154] also demonstrate an outstanding ability to scavenge DPPH free radicals [155]. Shahzadi et al. also observed the radical scavenging activity of bioinspired Co NPs and reported that the scavenging power and antioxidant activity are dose dependent [92]. Cobalt ferrite nanoparticles synthesized using *Monascus purpureus* cell-free culture filtrate exhibited a superparamagnetic nature according to the VSM analysis, and promising antioxidant activity compared to ascorbic acid as a standard according to the DPPH assay [146]. Cobalt nanomaterials can also aid in healing by increasing the number of fibrocyte, the concentration of hydroxyproline, hexuronic acid, hexosamine, and fibrocyte/fibroblast ratio [156]. Kulanthaivel et al. developed a highly efficient human mesenchymal stem cell (hMSC) encapsulation system by incorporating bivalent cobalt doped nano-hydroxyapatite (HAN) and gum tragacanth (GT) as angiogenic–osteogenic components into the calcium alginate (CA) beads, which could promote osteogenesis and angiogenesis [157]. Additionally, cobalt nanomaterials have other biomedical applications such as anticholinergic, and antidiabetic properties [84].

## 10. The Cytotoxicity of Cobalt Nanomaterials and Cobalt-Hybrid Nanomaterials

With the increasing number of applications of nanomaterials in various fields, such as food, cosmetics, and medicine, there is a significant concern about their safety [158]. The toxic effects of Co ions could be attributed to its competition with Ca(2+) in the cell-signaling and cell-binding proteins [159]. Cobalt has been shown to enter mitochondria, thereby inhibiting the respiratory chain. Additionally, cobalt has also been reported to show inhibition effects on the precursor processing of a single cytochrome c oxidase (COX) subunit and cytochrome c oxidase in mitochondria [160]. These results indicate the potential toxicity of excessive cobalt.

Cobalt nanomaterials and cobalt-hybrid nanomaterials also show the toxicity issues for biomedical application. A study has also shown that metal particles mainly composed of cobalt nanoparticles can cause systemic and local toxic reactions due to various physical and chemical factors [161]. Co_3_O_4_ NPs can enter cardiomyocytes to induce ROS production and DNA damage, and alter cellular electrophysiological and mechanical properties, leading to alterations in intracellular calcium handling and reduced electromechanical efficiency [162]. Exposure to Co NPs can cause oxidative stress, induce DNA damage and DNA mutation, and lead to lung inflammation and injury [163]. Apart from that, the fast-dissolving CoO NPs can also release cobalt ions to induce skin sensitization [164]. Some studies have also indicated the cytotoxicity of Co NPs against primary human dopaminergic neurons and platelets [165,166].

The toxicity of Co_3_O_4_ NP was recognized and presumably caused by the fast cell internalization through endocytosis via the clathrin-dependent pathway. Once inside the cells, Co_3_O_4_ NPs are preferably stored in endocytic vesicles and then recruited by lysosomes, whose acidic pH can progressively solubilize the NPs to continuously release cobalt ions [159]. After being released, the highly soluble Co(2+) can bind with synovial fluid proteins and adjacent tissue surfaces, followed by dissemination into the peripheral blood [4], and induce ROS production, DNA damage, and chromosomal aberration across cellular barriers [55]. Co NPs and Co salt triggered a dose-dependent cytotoxicity with the increase in cytosolic calcium, lipid peroxidation, and depletion of glutathione (GSH), and also suppressed glutathione peroxidase 4 (GPX4) mRNA and protein expression [159]. Moreover, Co NPs could induce ferroptosis-like cell death through the enhancement of intracellular reactive oxygen species (ROS) level, cytoplasmic Fe(2+) level, lipid peroxidation, and consumption of reduced GSH [167].

Although nanotechnology has many advantages and potential, the investigation of the interaction between NPs and biological systems is a major concern. It is crucial to further study the effects and detailed mechanisms of Co NPs-mediated cytotoxicity and explore effective methods for detoxification.

## 11. Challenges, Future Opportunities, and Perspectives

Cobalt, as a component of vitamin B12, participates in the metabolism of the human body by regulating multiple signaling events. Cobalt can stimulate hematopoiesis, enhance the immune responses, and show attractive antibacterial activities. Due to the unique physical, chemical, and biological properties, nanomaterials have shown advancing application potential in different areas. Using the advantages of cobalt and nanotechnology, cobalt nanomaterials have been developed to show some promising properties, including anticancer, anti-infection, and immunological regulation effects.

To develop nanomaterials into biomedical uses, their cytotoxicity is regarded as one of the most important issues. Cobalt nanomaterials exhibit a safe nature towards the normal cells and have no harmful effects in the human blood stream, which allow them to have a wide range of biological and medical application. Interestingly, other works as well as our previous works have both demonstrated that cobalt nanoparticles show very low cytotoxicity against normal cells, while the same dosages of cobalt nanoparticles show strong inhibition effects against cancer cells [93,94]. Our work also indicated that cobalt nanoparticles treatment did not induce any toxicity in mice, indicating their in vivo biocompatibility [94]. These results strongly suggest the safety of cobalt nanoparticles for further biomedical application; however, more systemic works are still needed to further evaluate their potential in vitro and in vivo toxicity. There is urgent need for long-term and real-time assessments of the pharmacological and pharmacokinetics of cobalt nanoparticles.

Traditionally, NPs are synthesized by either physical or chemical methods, which leads to environmental toxicity and energy-intensive labor. Cobalt nanomaterials, synthesized by a green route using the extracts of different plants, microorganisms, and other biological molecules are environmentally friendly, facile in terms of synthesis, low in cost, and are expected to provide maximum protection to human health. Based on the potential role in various therapies of cobalt nanomaterials, the detailed mechanisms for the biological activities of cobalt nanomaterials need to be further investigated. Moreover, the toxicity of cobalt nanomaterials also need to be further systemically explored in in vitro and in vivo models to establish their application strategy with limited side effects.

Due to the threatening facts of infectious disease and drug-resistance issues worldwide, it is crucial to develop more effective treatments against virus, bacteria, and fungi infections. Cobalt nanoparticles have been proven to show inhibition effects against different kinds of viruses, bacteria, and fungi, which suggests their strong potential to serv as novel anti-infectious agents. The surfaces of cobalt nanoparticles can interact directly with the bacterial outer membrane, causing the membrane damage or leading to inhibition of enzyme biosynthesis to destroy the bacteria functions and growth. However, more precise mechanisms of how cobalt nanoparticles inhibit or kill different pathogens remains to be further explored.

Most importantly, it is difficult for microbial pathogens to develop resistance towards cobalt nanomaterials. With the advantages of their optimal and potent antibacterial activity and proper stability, cobalt-based nanosystems are expected to not only provide novel possibilities against the drug-resistant mutants, but also avoid the emergence of drug resistance.

Cobalt nanomaterials can also serve as drug carriers for targeted drug delivery. The encapsulation of drugs into cobalt nanomaterials can not only reduce the drug toxicity, but can also improve the targeting effects of drugs to achieve enhanced anti-infectious efficiency of drugs. However, the targeting effects of cobalt nanomaterials still remain an unsolved issue that need more attention. Thus, the involvement of more chemists to prepare functional cobalt nanomaterials, or to perform surface modification of cobalt nanomaterials is critical to develop cobalt nanomaterials with high targeting effects. Moreover, the drug loading efficiency and controlled drug release behaviors of cobalt nanomaterials also need to be further improved, which could benefit the targeted drug delivery for more effective pathogen clearance.

Moreover, cobalt nanomaterials can also enhance immunization efficacy as a cargo of immunogens for modulating immune responses to kill the pathogenic microorganisms. These properties allow cobalt nanoparticles the possibility to act as anti-infectious immunomodulators, vaccine carriers, or vaccine adjuvants. Cobalt nanoparticles have been proven to activate the innate immunity for enhanced anti-infectious immunological responses, but their exact mechanisms and their effects on adaptive immunity remain to be further investigated. The combining of cobalt nanoparticles as immunomodulators and antibiotics is expected to show enhanced anti-infectious efficiency, which still need further investigation. With the advantages of selective lymph node accumulation, antigen assembly, and antigen presentation, cobalt nanomaterials can also be used for vaccine delivery by loading different kinds of antigens, such as proteins and RNAs. Furthermore, as a kind of novel innate immunity activation agents, cobalt nanoparticles also show the potential of adjuvants to enhance the immunological responses of vaccines. These immunological application for cobalt nanoparticles all require the in-depth exploration of their precise immunological regulation responses.

In addition, cobalt nanomaterials offer the potential for various biomedical applications regarding cancer therapy, parasitic resistance, antioxidant effects, and wound healing. Current advances in nanomaterials engineering indicate that well-designed nanomaterials have the potential to improve healthcare in the future. Therefore, further studies are needed to increase the understanding of the functional mechanisms and physical and chemical properties of cobalt nanomaterials so that they can be utilized in a variety of diseased conditions.

Additionally, in order to accelerate the development of cobalt nanomaterials for future clinical application, there is also a critical issue remains to be solved. Each of the biological activities of cobalt nanoparticles has different levels of sensitivity based on their different particle structures, sizes, and surface coatings. To ensure the future uses of cobalt nanoparticles, the basic issue requires the preparation of functional cobalt nanoparticles with quantified standards, which would allow the standardized application of cobalt nanoparticles.

## 12. Conclusions

Here, we have reviewed the recent trends and understanding for the synthesis, biotoxicity, and biological application of cobalt nanomaterials, in particular, discussing their anti-infective effects, mechanisms, and application. Overall, although a number of issues, such as their biological mechanisms and unexpected toxicity, are still needed to be clarified and solved, the promising abilities of cobalt nanomaterials indeed present an attractive prospect for further biological and medical uses, including anti-infectious application. With the increasing attention paid to cobalt nanomaterials, we believe that more strategies will be developed for anti-infection treatments based on cobalt nanomaterials, which would finally benefit the control of infectious diseases.

## Data Availability

Not applicable.

## References

[1] Guo Y., Song G., Sun M., Wang J., Wang Y. (2020). Prevalence and Therapies of Antibiotic-Resistance in *Staphylococcus aureus*. Front. Cell. Infect. Microbiol..

[2] Kollef M.H., Shorr A.F., Bassetti M., Timsit J.F., Micek S.T., Michelson A.P., Garnacho-Montero J. (2021). Timing of antibiotic therapy in the ICU. Crit. Care.

[3] Frieri M., Kumar K., Boutin A. (2017). Antibiotic resistance. J. Infect. Public Health.

[4] Leyssens L., Vinck B., Van Der Straeten C., Wuyts F., Maes L. (2017). Cobalt toxicity in humans—A review of the potential sources and systemic health effects. Toxicology.

[5] González-Montaña J.R., Escalera-Valente F., Alonso A.J., Lomillos J.M., Robles R., Alonso M.E. (2020). Relationship between Vitamin B12 and Cobalt Metabolism in Domestic Ruminant: An Update. Animals.

[6] Wang Z., Yan Y., Wang Y., Su Y., Qiao L. (2020). Lifecycle of cobalt-based alloy for artificial joints: From bulk material to nanoparticles and ions due to bio-tribocorrosion. J. Mater. Sci. Technol..

[7] Mukalel A.J., Riley R.S., Zhang R., Mitchell M.J. (2019). Nanoparticles for nucleic acid delivery: Applications in cancer immunotherapy. Cancer Lett..

[8] Song J., Liao L., Zhang Z., Yusran Y., Wang R., Fang J., Liu Y., Hou Y., Wang Y., Fang Q. (2022). 2D Microporous Covalent Organic Frameworks as Cobalt Nanoparticle Supports for Electrocatalytic Hydrogen Evolution Reaction. Crystals.

[9] Iravani S., Varma R. (2020). Sustainable synthesis of cobalt and cobalt oxide nanoparticles and their catalytic and biomedical applications. Green Chem..

[10] Francis W.R., Liu Z., Owens S.E., Wang X., Xue H., Lord A., Kanamarlapudi V., Xia Z. (2021). Role of hypoxia inducible factor 1alpha in cobalt nanoparticle induced cytotoxicity of human THP-1 macrophages. Biomater. Transl..

[11] Anwar A., Numan A., Siddiqui R., Khalid M., Khan N.A. (2019). Cobalt nanoparticles as novel nanotherapeutics against *Acanthamoeba castellanii*. Parasites Vectors.

[12] Sharma G., Kalra S.K., Tejan N., Ghoshal U. (2020). Nanoparticles based therapeutic efficacy against Acanthamoeba: Updates and future prospect. Exp. Parasitol..

[13] Turecka K., Chylewska A., Kawiak A., Waleron K.F. (2018). Antifungal Activity and Mechanism of Action of the Co(III) Coordination Complexes with Diamine Chelate Ligands Against Reference and Clinical Strains of *Candida* spp.. Front. Microbiol..

[14] Anwar A., Chi Fung L., Anwar A., Jagadish P., Numan A., Khalid M., Shahabuddin S., Siddiqui R., Khan N.A. (2019). Effects of Shape and Size of Cobalt Phosphate Nanoparticles against *Acanthamoeba castellanii*. Pathogens.

[15] Hu X., Wei X., Ling J., Chen J. (2021). Cobalt: An Essential Micronutrient for Plant Growth?. Front. Plant. Sci..

[16] Ammendola S., Ciavardelli D., Consalvo A., Battistoni A. (2020). Cobalt can fully recover the phenotypes related to zinc deficiency in *Salmonella* Typhimurium. Metallomics.

[17] Langan R.C., Goodbred A.J. (2017). Vitamin B12 Deficiency: Recognition and Management. Am. Fam. Physician.

[18] Roth W., Mohamadzadeh M. (2021). Vitamin B12 and gut-brain homeostasis in the pathophysiology of ischemic stroke. EBioMedicine.

[19] Obeid R., Heil S.G., Verhoeven M.M.A., van den Heuvel E., de Groot L., Eussen S. (2019). Vitamin B12 Intake From Animal Foods, Biomarkers, and Health Aspects. Front. Nutr..

[20] Fang H., Kang J., Zhang D. (2017). Microbial production of vitamin B(12): A review and future perspectives. Microb. Cell Fact..

[21] Weerathilake W., Brassington A.H., Williams S.J., Kwong W.Y., Sinclair L.A., Sinclair K.D. (2019). Added dietary cobalt or vitamin B12, or injecting vitamin B12 does not improve performance or indicators of ketosis in pre- and post-partum Holstein-Friesian dairy cows. Animal.

[22] Allen L.H., Miller J.W., de Groot L., Rosenberg I.H., Smith A.D., Refsum H., Raiten D.J. (2018). Biomarkers of Nutrition for Development (BOND): Vitamin B-12 Review. J. Nutr..

[23] Guéant J.L., Guéant-Rodriguez R.M., Alpers D.H. (2022). Vitamin B12 absorption and malabsorption. Vitam. Horm..

[24] Sugihara T., Koda M., Okamoto T., Miyoshi K., Matono T., Oyama K., Hosho K., Okano J.I., Isomoto H., Murawaki Y. (2017). Falsely Elevated Serum Vitamin B(12) Levels Were Associated with the Severity and Prognosis of Chronic Viral Liver Disease. Yonago Acta Med..

[25] Dror D.K., Allen L.H. (2018). Vitamin B-12 in Human Milk: A Systematic Review. Adv. Nutr..

[26] Wolffenbuttel B.H.R., Wouters H., Heiner-Fokkema M.R., van der Klauw M.M. (2019). The Many Faces of Cobalamin (Vitamin B12) Deficiency. Mayo Clin. Proc. Innov. Qual. Outcomes.

[27] Wang H., Li L., Qin L.L., Song Y., Vidal-Alaball J., Liu T.H. (2018). Oral vitamin B(12) versus intramuscular vitamin B(12) for vitamin B(12) deficiency. Cochrane Database Syst. Rev..

[28] Batista K.S., Cintra V.M., Lucena P.A.F., Manhaes-de-Castro R., Toscano A.E., Costa L.P., Queiroz M., de Andrade S.M., Guzman-Quevedo O., Aquino J.S. (2022). The role of vitamin B12 in viral infections: A comprehensive review of its relationship with the muscle-gut-brain axis and implications for SARS-CoV-2 infection. Nutr. Rev..

[29] Del Bo C., Riso P., Gardana C., Brusamolino A., Battezzati A., Ciappellano S. (2019). Effect of two different sublingual dosages of vitamin B(12) on cobalamin nutritional status in vegans and vegetarians with a marginal deficiency: A randomized controlled trial. Clin. Nutr..

[30] Green R. (2017). Vitamin B12 deficiency from the perspective of a practicing hematologist. Blood.

[31] Chen Y., Huang H., He X., Duan W., Mo X. (2021). Sex differences in the link between blood cobalt concentrations and insulin resistance in adults without diabetes. Environ. Health Prev. Med..

[32] Danzeisen R., Williams D.L., Viegas V., Dourson M., Verberckmoes S., Burzlaff A. (2020). Bioelution, Bioavailability, and Toxicity of Cobalt Compounds Correlate. Toxicol. Sci..

[33] Tjong E., Dimri M., Mohiuddin S.S. (2022). Biochemistry, Tetrahydrofolate. StatPearls.

[34] Chen R., Jiang T., She Y., Xu J., Li C., Zhou S., Shen H., Shi H., Liu S. (2017). Effects of Cobalt Chloride, a Hypoxia-Mimetic Agent, on Autophagy and Atrophy in Skeletal C2C12 Myotubes. BioMed Res. Int..

[35] Skalny A.V., Zaitseva I.P., Gluhcheva Y.G., Skalny A.A., Achkasov E.E., Skalnaya M.G., Tinkov A.A. (2019). Cobalt in athletes: Hypoxia and doping—New crossroads. J. Appl. Biomed..

[36] Ssempijja F., Iceland Kasozi K., Daniel Eze E., Tamale A., Ewuzie S.A., Matama K., Ekou J., Bogere P., Mujinya R., Musoke G.H. (2020). Consumption of Raw Herbal Medicines Is Associated with Major Public Health Risks amongst Ugandans. J. Environ. Public Health.

[37] Abass A.A., Abdulridha W.A.M., Alaarage W.K., Abdulrudha N.H., Haider J. (2021). Evaluating the antibacterial effect of cobalt nanoparticles against multi-drug resistant pathogens. J. Med. Life.

[38] Kumar R., Badogu K., Kour K., Farooq S., Singh R. (2022). Hydrogel-Nanofiber Composites for Tissue Reconstruction Applications: A State of the Art Review. Encycl. Mater. Plast. Polym..

[39] Bhattacharjee A., Hassan R., Gupta A., Verma M., Murugan P.A., Sengupta P., Matheshwaran S., Manna I., Balani K. (2020). Effect of Zn and Co Doping on Antibacterial Efficacy and Cytocompatibility of Spark Plasma Sintered Hydroxyapatite. J. Am. Ceram. Soc..

[40] Zhang E., Zhao X., Hu J., Wang R., Fu S., Qin G. (2021). Antibacterial metals and alloys for potential biomedical implants. Bioact. Mater..

[41] Kumar V., Mishra R.K., Kaur G., Dutta D. (2017). Cobalt and nickel impair DNA metabolism by the oxidative stress independent pathway. Metallomics.

[42] Fernandes L.P., Silva J.M.B., Martins D.O.S., Santiago M.B., Martins C.H.G., Jardim A.C.G., Oliveira G.S., Pivatto M., Souza R.A.C., Franca E.F. (2020). Fragmentation Study, Dual Anti-Bactericidal and Anti-Viral Effects and Molecular Docking of Cobalt(III) Complexes. Int. J. Mol. Sci..

[43] Díez-Tercero L., Delgado L.M., Bosch-Rué E., Perez R.A. (2021). Evaluation of the immunomodulatory effects of cobalt, copper and magnesium ions in a pro inflammatory environment. Sci. Rep..

[44] Maggini S., Pierre A., Calder P.C. (2018). Immune Function and Micronutrient Requirements Change over the Life Course. Nutrients.

[45] Wu D., Lewis E.D., Pae M., Meydani S.N. (2018). Nutritional Modulation of Immune Function: Analysis of Evidence, Mechanisms, and Clinical Relevance. Front. Immunol..

[46] Wang C., Zhang R., Wei X., Lv M., Jiang Z. (2020). Metalloimmunology: The metal ion-controlled immunity. Adv. Immunol..

[47] Luo Y., Fu Y., Huang Z., Li M. (2021). Transition metals and metal complexes in autophagy and diseases. J. Cell. Physiol..

[48] Yu Y., Li W., Ren L., Yang C., Li D., Han X., Sun Y., Lv C., Han F. (2018). Inhibition of autophagy enhanced cobalt chloride-induced apoptosis in rat alveolar type II epithelial cells. Mol. Med. Rep..

[49] Salloum Z., Lehoux E.A., Harper M.E., Catelas I. (2021). Effects of cobalt and chromium ions on glycolytic flux and the stabilization of hypoxia-inducible factor-1alpha in macrophages in vitro. J. Orthop. Res..

[50] Turecka K., Chylewska A., Rychłowski M., Zakrzewska J., Waleron K. (2021). Antibacterial Activity of Co(III) Complexes with Diamine Chelate Ligands against a Broad Spectrum of Bacteria with a DNA Interaction Mechanism. Pharmaceutics.

[51] Jonitz-Heincke A., Sellin M.L., Seyfarth A., Peters K., Mueller-Hilke B., Fiedler T., Bader R., Klinder A. (2019). Analysis of Cellular Activity Short-Term Exposure to Cobalt and Chromium Ions in Mature Human Osteoblasts. Materials.

[52] Dolomatov S., Sataeva T.P., Zukow W. (2019). Modern aspects of regulatory, pathophysiological and toxic effects of cobalt ions during oral intake in the human body. Health Risk Anal..

[53] Salloum Z., Lehoux E.A., Harper M.E., Catelas I. (2018). Effects of cobalt and chromium ions on oxidative stress and energy metabolism in macrophages in vitro. J. Orthop. Res..

[54] Kassapidou M., Stenport V.F., Johansson C.B., Ostberg A.K., Johansson P.H., Hjalmarsson L. (2021). Inflammatory Response to Cobalt-Chromium Alloys Fabricated With Different Techniques. J. Oral Maxillofac. Res..

[55] Xu J., Yang J., Nyga A., Ehteramyan M., Moraga A., Wu Y., Zeng L., Knight M.M., Shelton J.C. (2018). Cobalt (II) ions and nanoparticles induce macrophage retention by ROS-mediated down-regulation of RhoA expression. Acta Biomater..

[56] Riedel F., Aparicio-Soto M., Curato C., Thierse H.J., Siewert K., Luch A. (2021). Immunological Mechanisms of Metal Allergies and the Nickel-Specific TCR-pMHC Interface. Int. J. Environ. Res. Public Health.

[57] Riedel F., Aparicio-Soto M., Curato C., Munch L., Abbas A., Thierse H.J., Peitsch W.K., Luch A., Siewert K. (2022). Unique and common TCR repertoire features of Ni^2+^-, Co^2+^-, and Pd^2+^-specific human CD154 + CD4+ T cells. Allergy.

[58] Baskey S.J., Lehoux E.A., Catelas I. (2017). Effects of cobalt and chromium ions on lymphocyte migration. J. Orthop. Res..

[59] Xu J., Nyga A., Li W., Zhang X., Gavara N., Knight M.M., Shelton J. (2018). Cobalt ions stimulate a fibrotic response through matrix remodelling, fibroblast contraction and release of pro-fibrotic signals from macrophages. Eur. Cells Mater..

[60] Tsui H.C., Decaesteker T., Jonckheere A.C., Vande Velde G., Cremer J., Verbeken E., Hoet P.H.M., Nemery B., Vanoirbeek J.A.J. (2020). Cobalt exposure via skin alters lung immune cells and enhances pulmonary responses to cobalt in mice. Am. J. Physiol. Lung Cell. Mol. Physiol..

[61] Esa Y.A., Sapawe N. (2020). A short review on biosynthesis of cobalt metal nanoparticles. Mater. Today Proc..

[62] Vishwanath R., Negi B. (2021). Conventional and green methods of synthesis of silver nanoparticles and their antimicrobial properties. Curr. Res. Green Sustain. Chem..

[63] Jamkhande P.G., Ghule N.W., Bamer A.H., Kalaskar M.G. (2019). Metal nanoparticles synthesis: An overview on methods of preparation, advantages and disadvantages, and applications. J. Drug Deliv. Sci. Technol..

[64] Vijayanandan A.S., Balakrishnan R.M. (2018). Biosynthesis of cobalt oxide nanoparticles using endophytic fungus *Aspergillus nidulans*. J. Environ. Manag..

[65] Khusnuriyalova A., Caporali M., Hey-Hawkins E., Sinyashin O., Yakhvarov D. (2021). Preparation of Cobalt Nanoparticles. Eur. J. Inorg. Chem..

[66] Qasim S., Zafar A., Saif M.S., Ali Z., Nazar M., Waqas M., Haq A.U., Tariq T., Hassan S.G., Iqbal F. (2020). Green synthesis of iron oxide nanorods using *Withania coagulans* extract improved photocatalytic degradation and antimicrobial activity. J. Photochem. Photobiol. B.

[67] Tripathi D., Modi A., Narayan G., Rai S.P. (2019). Green and cost effective synthesis of silver nanoparticles from endangered medicinal plant *Withania coagulans* and their potential biomedical properties. Mater. Sci. Eng. C Mater. Biol. Appl..

[68] Ahmad S., Munir S., Zeb N., Ullah A., Khan B., Ali J., Bilal M., Omer M., Alamzeb M., Salman S.M. (2019). Green nanotechnology: A review on green synthesis of silver nanoparticles—An ecofriendly approach. Int. J. Nanomed..

[69] Singh J., Dutta T., Kim K.H., Rawat M., Samddar P., Kumar P. (2018). ‘Green’ synthesis of metals and their oxide nanoparticles: Applications for environmental remediation. J. Nanobiotechnol..

[70] Singh A.K. (2022). A review on plant extract-based route for synthesis of cobalt nanoparticles: Photocatalytic, electrochemical sensing and antibacterial applications. Curr. Res. Green Sustain. Chem..

[71] Bibi I., Nazar N., Iqbal M., Kamal S., Nawaz H., Nouren S., Safa Y., Jilani K., Sultan M., Ata S. (2017). Green and eco-friendly synthesis of cobalt-oxide nanoparticle: Characterization and photo-catalytic activity. Adv. Powder Technol..

[72] Krishna P.G., Chandra Mishra P., Naika M.M., Gadewar M., Ananthaswamy P.P., Rao S., Boselin Prabhu S.R., Yatish K.V., Nagendra H.G., Moustafa M. (2022). Photocatalytic Activity Induced by Metal Nanoparticles Synthesized by Sustainable Approaches: A Comprehensive Review. Front. Chem..

[73] Manzanares D., Ceña V. (2020). Endocytosis: The Nanoparticle and Submicron Nanocompounds Gateway into the Cell. Pharmaceutics.

[74] Jahan S.T., Sadat S.M.A., Walliser M., Haddadi A. (2017). Targeted Therapeutic Nanoparticles: An Immense Promise to Fight against Cancer. J. Drug Deliv..

[75] de la Harpe K.M., Kondiah P.P.D., Choonara Y.E., Marimuthu T., du Toit L.C., Pillay V. (2019). The Hemocompatibility of Nanoparticles: A Review of Cell-Nanoparticle Interactions and Hemostasis. Cells.

[76] Janagam D.R., Wu L., Lowe T.L. (2017). Nanoparticles for drug delivery to the anterior segment of the eye. Adv. Drug Deliv. Rev..

[77] Yuan D., He H., Wu Y., Fan J., Cao Y. (2019). Physiologically Based Pharmacokinetic Modeling of Nanoparticles. J. Pharm. Sci..

[78] Dzulkharnien N.S.F., Rohani R. (2022). A Review on Current Designation of Metallic Nanocomposite Hydrogel in Biomedical Applications. Nanomaterials.

[79] Lin S., Dong J., Zhang B., Yuan Z., Lu C., Han P., Xu J., Jia L., Wang L. (2021). Synthesis of bifunctional fluorescent nanohybrids of carbon dots-copper nanoclusters via a facile method for Fe^3+^ and Tb^3+^ ratiometric detection. Anal. Methods.

[80] Han X., Xu K., Taratula O., Farsad K. (2019). Applications of nanoparticles in biomedical imaging. Nanoscale.

[81] Islam T., Hasan M.M., Awal A., Nurunnabi M., Ahammad A.J.S. (2020). Metal Nanoparticles for Electrochemical Sensing: Progress and Challenges in the Clinical Transition of Point-of-Care Testing. Molecules.

[82] Sondhi P., Maruf M.H.U., Stine K.J. (2019). Nanomaterials for Biosensing Lipopolysaccharide. Biosensors.

[83] Arikan K., Burhan H., Bayat R., Sen F. (2022). Glucose nano biosensor with non-enzymatic excellent sensitivity prepared with nickel-cobalt nanocomposites on f-MWCNT. Chemosphere.

[84] Waris A., Din M., Ali A., Afridi S., Baset A., Khan A.U., Ali M. (2021). Green fabrication of Co and Co_3_O_4_ nanoparticles and their biomedical applications: A review. Open Life Sci..

[85] Rozina, Ahmad M., Alruqi M., Zafar M. (2022). Cleaner production of biodiesel from novel and non-edible seed oil of *Chamaerops humilis* using recyclable cobalt oxide nanoparticles: A contribution to resilient and sustainable world. J. Clean. Prod..

[86] Maksoud M., El-Sayyad G.S., Ashour A.H., El-Batal A.I., Elsayed M.A., Gobara M., El-Khawaga A.M., Abdel-Khalek E.K., El-Okr M.M. (2019). Antibacterial, antibiofilm, and photocatalytic activities of metals-substituted spinel cobalt ferrite nanoparticles. Microb. Pathog..

[87] Rauwel E., Al-Arag S., Salehi H., Amorim C.O., Cuisinier F., Guha M., Rosario M.S., Rauwel P. (2020). Assessing Cobalt Metal Nanoparticles Uptake by Cancer Cells Using Live Raman Spectroscopy. Int. J. Nanomed..

[88] Khannanov A.A., Rossova A.A., Ignatyeva K.A., Ulakhovich N.A., Gerasimov A.V., Boldyrev A.E., Evtugyn V.G., Rogov A.M., Cherosov M.A., Gilmutdinov I.F. (2022). Superparamagnetic cobalt nanoparticles in hyperbranched polyester polyol matrix with anti-protease activity. J. Magn. Magn. Mater..

[89] Farkas B., Terranova U., de Leeuw N.H. (2020). Binding modes of carboxylic acids on cobalt nanoparticles. Phys. Chem. Chem. Phys..

[90] Kong I.C., Ko K.S., Koh D.C., Chon C.M. (2020). Comparative Effects of Particle Sizes of Cobalt Nanoparticles to Nine Biological Activities. Int. J. Mol. Sci..

[91] Nagababu U., Shanmukha Kumar J.V., Rafi Shaik M., Sharaf M.A.F. (2021). Facile synthesis, physiochemical characterization and bio evaluation of sulfadimidine capped cobalt nanoparticles. Saudi J. Biol. Sci..

[92] Shahzadi T., Zaib M., Riaz T., Shehzadi S., Abbasi M., Shahid M. (2019). Synthesis of Eco-friendly Cobalt Nanoparticles Using Celosia argentea Plant Extract and Their Efficacy Studies as Antioxidant, Antibacterial, Hemolytic and Catalytical Agent. Arab. J. Sci. Eng..

[93] Ansari S.M., Bhor R.D., Pai K.R., Sen D., Mazumder S., Ghosh K., Kolekar Y.D., Ramana C.V. (2017). Cobalt nanoparticles for biomedical applications: Facile synthesis, physiochemical characterization, cytotoxicity behavior and biocompatibility. Appl. Surf. Sci..

[94] Huang X., Cai H., Zhou H., Li T., Jin H., Evans C.E., Cai J., Pi J. (2021). Cobalt oxide nanoparticle-synergized protein degradation and phototherapy for enhanced anticancer therapeutics. Acta Biomater..

[95] Andleeb A., Andleeb A., Asghar S., Zaman G., Tariq M., Mehmood A., Nadeem M., Hano C., Lorenzo J.M., Abbasi B.H. (2021). A Systematic Review of Biosynthesized Metallic Nanoparticles as a Promising Anti-Cancer-Strategy. Cancers.

[96] Akhtar M.J., Ahamed M., Alhadlaq H.A., Alshamsan A. (2017). Nanotoxicity of cobalt induced by oxidant generation and glutathione depletion in MCF-7 cells. Toxicol. Vitr..

[97] Fu Z., Xiang J. (2020). Aptamer-Functionalized Nanoparticles in Targeted Delivery and Cancer Therapy. Int. J. Mol. Sci..

[98] La-Beck N.M., Islam M.R., Markiewski M.M. (2020). Nanoparticle-Induced Complement Activation: Implications for Cancer Nanomedicine. Front. Immunol..

[99] Mukherjee A., Paul M., Mukherjee S. (2019). Recent Progress in the Theranostics Application of Nanomedicine in Lung Cancer. Cancers.

[100] Zhao J., Liu Y., Sun J., Zhu H., Chen Y., Dong T., Sang R., Gao X., Yang W., Deng Y. (2020). Magnetic targeting cobalt nanowire-based multifunctional therapeutic system for anticancer treatment and angiogenesis. Colloids Surf. B Biointerfaces.

[101] Zhu H., Deng J., Yang Y., Li Y., Shi J., Zhao J., Deng Y., Chen X., Yang W. (2019). Cobalt nanowire-based multifunctional platform for targeted chemo-photothermal synergistic cancer therapy. Colloids Surf. B Biointerfaces.

[102] Tombuloglu H., Khan F.A., Almessiere M.A., Aldakheel S., Baykal A. (2021). Synthesis of niobium substituted cobalt-nickel nano-ferrite (Co_0.5_Ni_0.5_Nb_x_Fe_2-x_O_4_ (x ≤ 0.1) by hydrothermal approach show strong anti-colon cancer activities. J. Biomol. Struct. Dyn..

[103] Jarestan M., Khalatbari K., Pouraei A., Sadat Shandiz S.A., Beigi S., Hedayati M., Majlesi A., Akbari F., Salehzadeh A. (2020). Preparation, characterization, and anticancer efficacy of novel cobalt oxide nanoparticles conjugated with thiosemicarbazide. 3 Biotech.

[104] Bejarbaneh M., Moradi-Shoeili Z., Jalali A., Salehzadeh A. (2020). Synthesis of Cobalt Hydroxide Nano-flakes Functionalized with Glutamic Acid and Conjugated with Thiosemicarbazide for Anticancer Activities Against Human Breast Cancer Cells. Biol. Trace Elem. Res..

[105] Vimbela G.V., Ngo S.M., Fraze C., Yang L., Stout D.A. (2017). Antibacterial properties and toxicity from metallic nanomaterials. Int. J. Nanomed..

[106] Tacconelli E., Carrara E., Savoldi A., Harbarth S., Mendelson M., Monnet D.L., Pulcini C., Kahlmeter G., Kluytmans J., Carmeli Y. (2018). Discovery, research, and development of new antibiotics: The WHO priority list of antibiotic-resistant bacteria and tuberculosis. Lancet Infect. Dis..

[107] Mohammadi F., Gholami A., Omidifar N., Amini A., Kianpour S., Taghizadeh S.M. (2022). The potential of surface nano-engineering in characteristics of cobalt-based nanoparticles and biointerface interaction with prokaryotic and human cells. Colloids Surf. B Biointerfaces.

[108] Kharade S., Nikam G., Mane-Gavade S., Patil S., Gaikwad K. (2020). Biogenic synthesis of Cobalt nanoparticles using Hibiscus cannabinus leaf extract and their antibacterial activity. Res. J. Chem. Environ..

[109] Hafeez M., Shaheen R., Akram B., Zain-ul A., Haq S., Mahsud S., Ali S., Khan R.T. (2020). Green synthesis of cobalt oxide nanoparticles for potential biological applications. Mater. Res. Express.

[110] Pageni P., Yang P., Chen Y.P., Huang Y., Bam M., Zhu T., Nagarkatti M., Benicewicz B.C., Decho A.W., Tang C. (2018). Charged Metallopolymer-Grafted Silica Nanoparticles for Antimicrobial Applications. Biomacromolecules.

[111] Lan S., Zhang J., Li X., Pan L., Li J., Wu X., Yang S.T. (2022). Low Toxicity of Metal-Organic Framework MOF-74(Co) Nano-Particles In Vitro and In Vivo. Nanomaterials.

[112] Li Z., Ma J., Ruan J., Zhuang X. (2019). Using Positively Charged Magnetic Nanoparticles to Capture Bacteria at Ultralow Concentration. Nanoscale Res. Lett..

[113] Rethinasabapathy M., Vilian A.T.E., Hwang S.K., Kang S.-M., Cho Y., Han Y.-K., Rhee J.-K., Huh Y.S. (2021). Cobalt ferrite microspheres as a biocompatible anode for higher power generation in microbial fuel cells. J. Power Sources.

[114] Kong I.C., Ko K.S., Koh D.C. (2020). Evaluation of the Effects of Particle Sizes of Silver Nanoparticles on Various Biological Systems. Int. J. Mol. Sci..

[115] Baptista P.V., McCusker M.P., Carvalho A., Ferreira D.A., Mohan N.M., Martins M., Fernandes A.R. (2018). Nano-Strategies to Fight Multidrug Resistant Bacteria—“A Battle of the Titans”. Front. Microbiol..

[116] Khurana A., Tekula S., Saifi M.A., Venkatesh P., Godugu C. (2019). Therapeutic applications of selenium nanoparticles. Biomed. Pharmacother..

[117] Horvat S. (2021). Development of Nanocarriers for Treatment and Diagnostics of Aspergillosis. Doctoral Thesis.

[118] Tanvir F., Yaqub A., Tanvir S., Anderson W.A. (2017). Poly-L-arginine Coated Silver Nanoprisms and Their Anti-Bacterial Properties. Nanomaterials.

[119] Moradpoor H., Safaei M., Rezaei F., Golshah A., Jamshidy L., Hatam R., Abdullah R.S. (2019). Optimisation of Cobalt Oxide Nanoparticles Synthesis as Bactericidal Agents. Open Access Maced. J. Med. Sci..

[120] Hoseinzadeh E., Makhdoumi P., Taha P., Hossini H., Stelling J., Kamal M.A., Ashraf G.M. (2017). A Review on Nano-Antimicrobials: Metal Nanoparticles, Methods and Mechanisms. Curr. Drug Metab..

[121] Liu P., Wang Y., Han L., Cai Y., Ren H., Ma T., Li X., Petrenko V.A., Liu A. (2020). Colorimetric Assay of Bacterial Pathogens Based on Co_3_O_4_ Magnetic Nanozymes Conjugated with Specific Fusion Phage Proteins and Magnetophoretic Chromatography. ACS Appl. Mater. Interfaces.

[122] Sharmin S., Rahaman M.M., Sarkar C., Atolani O., Islam M.T., Adeyemi O.S. (2021). Nanoparticles as antimicrobial and antiviral agents: A literature-based perspective study. Heliyon.

[123] Nikaeen G., Abbaszadeh S., Yousefinejad S. (2020). Application of nanomaterials in treatment, anti-infection and detection of coronaviruses. Nanomedicine.

[124] Roy A., Datta S., Roy M., Alghamdi S., Rajab B.S., Babalghith A.O., Islam M.R. (2022). Nanomaterials and Bioactive Compounds against SARS-CoV-2. J. Nanomater..

[125] Vahedifard F., Chakravarthy K. (2021). Nanomedicine for COVID-19: The role of nanotechnology in the treatment and diagnosis of COVID-19. Emergent Mater..

[126] Chakravarty M., Vora A. (2021). Nanotechnology-based antiviral therapeutics. Drug Deliv. Transl. Res..

[127] Gurunathan S., Qasim M., Choi Y., Do J.T., Park C., Hong K., Kim J.H., Song H. (2020). Antiviral Potential of Nanoparticles-Can Nanoparticles Fight Against Coronaviruses?. Nanomaterials.

[128] DeLong R.K., Swanson R., Niederwerder M.C., Khanal P., Aryal S., Marasini R., Jaberi-Douraki M., Shakeri H., Mazloom R., Schneider S. (2021). Zn-based physiometacomposite nanoparticles: Distribution, tolerance, imaging, and antiviral and anticancer activity. Nanomedicine.

[129] Kevadiya B.D., Woldstad C., Ottemann B.M., Dash P., Sajja B.R., Lamberty B., Morsey B., Kocher T., Dutta R., Bade A.N. (2018). Multimodal Theranostic Nanoformulations Permit Magnetic Resonance Bioimaging of Antiretroviral Drug Particle Tissue-Cell Biodistribution. Theranostics.

[130] Rauch S., Jasny E., Schmidt K.E., Petsch B. (2018). New Vaccine Technologies to Combat Outbreak Situations. Front. Immunol..

[131] Edagwa B.J., Gendelman H.E. (2018). Antimicrobials: Broad-spectrum antivirals. Nat. Mater..

[132] Singh L., Kruger H.G., Maguire G.E.M., Govender T., Parboosing R. (2017). The role of nanotechnology in the treatment of viral infections. Ther. Adv. Infect. Dis..

[133] Milovanovic M., Arsenijevic A., Milovanovic J., Kanjevac T., Arsenijevic N., Grumezescu A.M. (2017). Chapter 14—Nanoparticles in Antiviral Therapy. Antimicrobial Nanoarchitectonics.

[134] Fries C.N., Curvino E.J., Chen J.L., Permar S.R., Fouda G.G., Collier J.H. (2021). Advances in nanomaterial vaccine strategies to address infectious diseases impacting global health. Nat. Nanotechnol..

[135] Gomes A.C., Mohsen M., Bachmann M.F. (2017). Harnessing Nanoparticles for Immunomodulation and Vaccines. Vaccines.

[136] Marques Neto L.M., Kipnis A., Junqueira-Kipnis A.P. (2017). Role of Metallic Nanoparticles in Vaccinology: Implications for Infectious Disease Vaccine Development. Front. Immunol..

[137] DeLong R.K., Comer J., Mathew E.N., Jaberi-Douraki M. (2019). Comparative Molecular Immunological Activity of Physiological Metal Oxide Nanoparticle and its Anticancer Peptide and RNA Complexes. Nanomaterials.

[138] Kannan P., Subramanian P., Maiyalagan T., Jiang Z. (2019). Cobalt Oxide Porous Nanocubes-Based Electrochemical Immunobiosensing of Hepatitis B Virus DNA in Blood Serum and Urine Samples. Anal. Chem..

[139] Azab S.M., Fekry A.M. (2017). Electrochemical design of a new nanosensor based on cobalt nanoparticles, chitosan and MWCNT for the determination of daclatasvir: A hepatitis C antiviral drug. RSC Adv..

[140] Vadlamani B.S., Uppal T., Verma S.C., Misra M. (2020). Functionalized TiO2 Nanotube-Based Electrochemical Biosensor for Rapid Detection of SARS-CoV-2. Sensors.

[141] Hall R.A., Noverr M.C. (2017). Fungal interactions with the human host: Exploring the spectrum of symbiosis. Curr. Opin. Microbiol..

[142] Strickland A.B., Shi M. (2021). Mechanisms of fungal dissemination. Cell. Mol. Life Sci..

[143] Al-Fakeh M.S., Alsaedi R.O. (2021). Synthesis, Characterization, and Antimicrobial Activity of CoO Nanoparticles from a Co (II) Complex Derived from Polyvinyl Alcohol and Aminobenzoic Acid Derivative. Sci. World J..

[144] Sharma P., Sharma A., Sharma M., Bhalla N., Estrela P., Jain A., Thakur P., Thakur A. (2017). Nanomaterial Fungicides: In Vitro and In Vivo Antimycotic Activity of Cobalt and Nickel Nanoferrites on Phytopathogenic Fungi. Glob. Chall..

[145] Rehman S., Almessiere M.A., Al-Jameel S.S., Ali U., Slimani Y., Tashkandi N., Al-Saleh N.S., Manikandan A., Khan F.A., Al-Suhaimi E.A. (2021). Designing of Co_0.5_Ni_0.5_Ga_x_Fe_2-x_O_4_ (0.0 ≤ x ≤ 1.0) Microspheres via Hydrothermal Approach and Their Selective Inhibition on the Growth of Cancerous and Fungal Cells. Pharmaceutics.

[146] El-Sayed E.R., Abdelhakim H.K., Zakaria Z. (2020). Extracellular biosynthesis of cobalt ferrite nanoparticles by *Monascus purpureus* and their antioxidant, anticancer and antimicrobial activities: Yield enhancement by gamma irradiation. Mater. Sci. Eng. C Mater. Biol. Appl..

[147] Fierascu I., Fierascu I.C., Brazdis R.I., Baroi A.M., Fistos T., Fierascu R.C. (2020). Phytosynthesized Metallic Nanoparticles-between Nanomedicine and Toxicology. A Brief Review of 2019’s Findings. Materials.

[148] Hasan M., Zafar A., Shahzadi I., Luo F., Hassan S.G., Tariq T., Zehra S., Munawar T., Iqbal F., Shu X. (2020). Fractionation of Biomolecules in *Withania coagulans* Extract for Bioreductive Nanoparticle Synthesis, Antifungal and Biofilm Activity. Molecules.

[149] Mishra V., Singh M., Mishra Y., Charbe N., Nayak P., Sudhakar K., Aljabali A., Shahcheraghi S., Bakshi H., Serrano-Aroca Á. (2021). Nanoarchitectures in Management of Fungal Diseases: An Overview. Appl. Sci..

[150] Li B., Pan L., Zhang H., Xie L., Wang X., Shou J., Qi Y., Yan X. (2021). Recent Developments on Using Nanomaterials to Combat *Candida albicans*. Front. Chem..

[151] Younus I., Khan S., Maqbool S., Begum Z. (2022). Anti-fungal therapy via incorporation of nanostructures: A systematic review for new dimensions. Phys. Scr..

[152] Pathakumari B., Liang G., Liu W. (2020). Immune defence to invasive fungal infections: A comprehensive review. Biomed. Pharmacother..

[153] Khalil A.T., Ovais M., Ullah I., Ali M., Shinwari Z.K., Maaza M. (2020). Physical properties, biological applications and biocompatibility studies on biosynthesized single phase cobalt oxide (Co_3_O_4_) nanoparticles via *Sageretia thea* (Osbeck.). Arab. J. Chem..

[154] Ghadi F., Ghara A., Naeimi A. (2018). Phytochemical fabrication, characterization, and antioxidant application of copper and cobalt oxides nanoparticles using *Sesbania sesban* plant. Chem. Pap..

[155] Alhujaily M., Albukhaty S., Yusuf M., Mohammed M.K.A., Sulaiman G., Alkaragoly H., Alyamani A., Albaqami J., Almalki F. (2022). Recent Advances in Plant-Mediated Zinc Oxide Nanoparticles with Their Significant Biomedical Properties. Bioengineering.

[156] Shalaby M.A., Anwar M.M., Saeed H. (2022). Nanomaterials for application in wound Healing: Current state-of-the-art and future perspectives. J. Polym. Res..

[157] Kulanthaivel S., Agarwal T., Sharan Rathnam V.S., Pal K., Banerjee I. (2021). Cobalt doped nano-hydroxyapatite incorporated gum tragacanth-alginate beads as angiogenic-osteogenic cell encapsulation system for mesenchymal stem cell based bone tissue engineering. Int. J. Biol. Macromol..

[158] Yoshioka Y., Kuroda E., Hirai T., Tsutsumi Y., Ishii K.J. (2017). Allergic Responses Induced by the Immunomodulatory Effects of Nanomaterials upon Skin Exposure. Front. Immunol..

[159] Borgese M., Rossi F., Bonfanti P., Colombo A., Mantecca P., Valdatta L., Bernardini G., Gornati R. (2020). Recovery ability of human adipose stem cells exposed to cobalt nanoparticles: Outcome of dissolution. Nanomedicine.

[160] Chamaon K., Schönfeld P., Awiszus F., Bertrand J., Lohmann C.H. (2019). Ionic cobalt but not metal particles induces ROS generation in immune cells in vitro. J. Biomed. Mater. Res. B Appl. Biomater..

[161] Zhang W., Wang C., Zhu W., Liu F., Liu Y. (2022). Ferrostatin-1 alleviates cytotoxicity of cobalt nanoparticles by inhibiting ferroptosis. Bioengineered.

[162] Savi M., Bocchi L., Cacciani F., Vilella R., Buschini A., Perotti A., Galati S., Montalbano S., Pinelli S., Frati C. (2021). Cobalt oxide nanoparticles induce oxidative stress and alter electromechanical function in rat ventricular myocytes. Part. Fibre Toxicol..

[163] Wan R., Mo Y., Zhang Z., Jiang M., Tang S., Zhang Q. (2017). Cobalt nanoparticles induce lung injury, DNA damage and mutations in mice. Part. Fibre Toxicol..

[164] Kim S.H., Lee J.H., Jung K., Yang J.Y., Shin H.S., Lee J.P., Jeong J., Oh J.H., Lee J.K. (2021). Copper and Cobalt Ions Released from Metal Oxide Nanoparticles Trigger Skin Sensitization. Front. Pharmacol..

[165] Gupta G., Gliga A., Hedberg J., Serra A., Greco D., Odnevall Wallinder I., Fadeel B. (2020). Cobalt nanoparticles trigger ferroptosis-like cell death (oxytosis) in neuronal cells: Potential implications for neurodegenerative disease. FASEB J..

[166] Taterra D., Skinningsrud B., Pękala P.A., Tomaszewska I.M., Marycz K., Radomski M.W., Tomaszewski K.A. (2021). In vitro effects of cobalt and chromium nanoparticles on human platelet function. Nanotoxicology.

[167] Liu Y., Zhu W., Ni D., Zhou Z., Gu J.H., Zhang W., Sun H., Liu F. (2020). Alpha lipoic acid antagonizes cytotoxicity of cobalt nanoparticles by inhibiting ferroptosis-like cell death. J. Nanobiotechnol..

